# Metabolic engineering of *Escherichia coli* to high efficient synthesis phenylacetic acid from phenylalanine

**DOI:** 10.1186/s13568-017-0407-0

**Published:** 2017-05-25

**Authors:** Lihua Zhang, Qian Liu, Hong Pan, Xun Li, Daoyi Guo

**Affiliations:** 1 College of Life and Environmental Sciences, Gannan Normal University, Ganzhou, 341000 People’s Republic of China; 2 Key Laboratory of Organo-Pharmaceutical Chemistry, Jiangxi Province, Gannan Normal University, Ganzhou, 341000 People’s Republic of China

**Keywords:** Metabolic engineering, Phenylacetic acid, Phenylalanine, Phenylacetaldehyde

## Abstract

Phenylacetic acid (PAA) is a fine chemical with a high industrial demand for its widespread uses. Whereas, microorganic synthesis of PAA is impeded by the formation of by-product phenethyl alcohol due to quick, endogenous, and superfluous conversion of aldehydes to their corresponding alcohols, which resulted in less conversation of PAA from aldehydes. In this study, an *Escherichia coli* K-12 MG1655 strain with reduced aromatic aldehyde reduction (RARE) that does duty for a platform for aromatic aldehyde biosynthesis was used to prompt more PAA biosynthesis. We establish a microbial biosynthetic pathway for PAA production from the simple substrate phenylalanine in *E. coli* with heterologous coexpression of aminotransferase (ARO8), keto acid decarboxylase (KDC) and aldehyde dehydrogenase H (AldH) gene. It was found that PAA transformation yield was up to ~94% from phenylalanine in *E. coli* and there was no by-product phenethyl alcohol was detected. Our results reveal the high efficiency of the RARE strain for production of PAA and indicate the potential industrial applicability of this microbial platform for PAA biosynthesis.

## Introduction

Phenylacetic acid (PAA) has received much attention on account of its extensive applications, which offer the huge demand. It has lots of applicable uses in medicine, pesticides, disinfectants and other industries (Dongamanti et al. 2012; Duan et al. 2000; Huang et al. 2014a), and has also been investigated as a kind of industrial raw material. Nowadays it is procured mainly by chemical methods. PAA could be obtained by chemical synthesis from different substrates like benzyl chloride, benzyl cyanide, mandelic acid, or ethylbenzene (Giroux et al. 2000; Milne et al. 2012). However, the methods of producing PAA via chemical synthesis have many drawbacks. The substrates such as sodium cyanide and benzyl cyanide are poisonous substances which are harmful to environment and the operation personnel. Although there are some strategies using enzyme catalytic synthesis of PAA, for example utilizing a nitrile hydratase and an amidase of *Rhodococcus equi* TG328 (Gilligan et al. 1993) or an arylacetonitrilase from *Pseudomonas fluorescens* EBC191 (Sosedov et al. 2010), the yield is low or the cost is high.

Biosynthesis in microbe cell factory has many advantages, compared with chemical synthesis or in vitro enzyme catalytic synthesis (Agapakis et al. 2012; Huang et al. 2014b). Nowadays, microbes are employed for the production of a wide array of complex drug molecules or precursors (Chen and Nielsen 2013; Nielsen et al. 2014), such as biofuel molecule (Rabinovitch-Deere et al. 2013), limonene and perillyl alcohol (Alonso-Gutierrez et al. 2013), terpenoids (Gupta et al. 2015; Wang et al. 2016), l-methionine (Huang et al. 2016). PAA is derived from the amino acid Phe through the intermediate phenylpyruvate in fungi and bacteria (Kishore et al. 1976; Krings et al. 1996; Groot and Bont 1998). Recently there is an observation suggesting that transamination of phenylalanine, decarboxylation of phenylpyruvate, subsequent oxidation of phenylacetaldehyde would be the most likely pathway for PAA synthesis (Cook et al. 2016; Somers et al. 2005). Aminotransferase (ARO8) and keto acid decarboxylase (KDC) have been shown to catalyze the first and the second steps in *Saccharomyces cerevisiae* (Li et al. 2016). However, aldehyde dehydrogenase involved in the last step to generate PAA has not yet been experimentally characterized and this knowledge gap limits the process development for producing PAA from phenylalanine. Aldehyde dehydrogenase are a superfamily enzymes which catalyze the oxidation of a large variety of aldehydes (Jo et al. 2008). It was reported previously that FeaB, AldB and AldH from *E. coli* were characterized as aldehyde dehydrogenase activity for the oxidation of phenylacetaldehyde or benzaldehyde in vitro (Ho and Weiner 2005; Jo et al. 2008; Koma et al. 2012). Therefore, we assessed these aldehyde dehydrogenases such as FeaB, AldB and AldH for biosynthesis of PAA from phenylalanine in the intracellular.

**Fig. 1:**
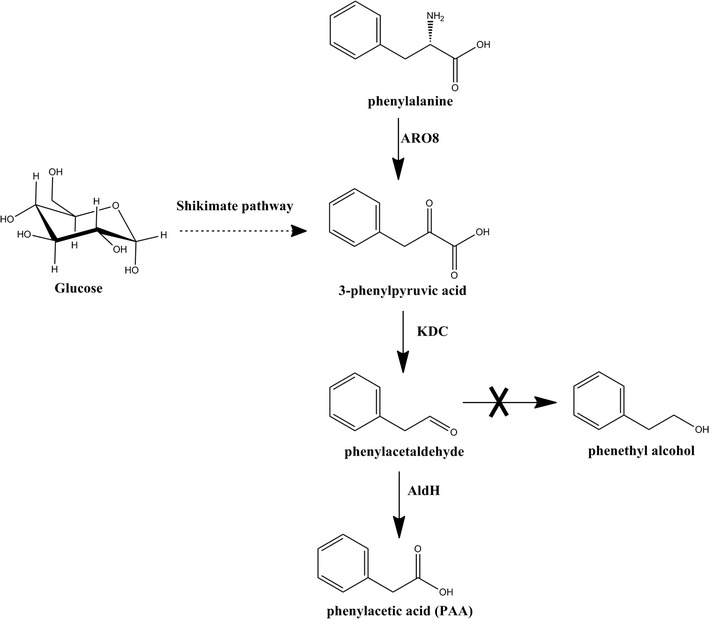
The phenylacetic acid (PAA) biosynthesis pathway used in this study

In this study, we establish a microbial biosynthetic pathway for PAA production from the simple substrate phenylalanine through overexpression of an aminotransferase gene ARO8, a keto acid decarboxylase gene KDC from *S. cerevisiae* and an aldehyde dehydrogenase H gene aldH from *E. coli* in *E. coli* (Fig. 1). Phenylacetaldehyde can be reduced to 2-phenylethanol that compets with PAA (Fig. 1), so we further assessed a reduced aromatic aldehyde reduction (RARE) *E. coli* K-12 MG1655 strain for biosynthesis of PAA (Kunjapur et al. 2014). It showed about 94% molar transformation yield from phenylalanine in this strain, which demonstrates the potential industrial applicability of this microbial platform for PAA biosynthesis.

## Materials and methods

### Materials

Reagents and solvents purchased from Sigma-Aldrich. Restriction enzymes, T4 DNA ligase and DNA polymerase were purchased from New England Biolabs and used according to the manufacturer’s specifications. Plasmid mini kits, PCR purification kits and gel extraction kits were ordered from Fermentas (Burlington, Canada) and used according to the manufacturer’s specifications. DNA primers were synthesized by GenScript, Nanjing, China.

### Plasmid construction in this research work

A plasmid of pDG30 for expression KDC gene (GenBank: NP_010668.3) from *S. cerevisiae* YPH499 was constructed in our previous study (Guo et al. 2017). The ARO8 (GenBank: EWH18548.1) gene was amplified by PCR from *S. cerevisiae* YPH499 genomic DNA using primers ARO8-*Xba*I and ARO8-*Xho*I, and inserted into pET28a(+) to give pDG2. The phenylacetaldehyde dehydrogenase (FeaB, GenBank: 945933), aldehyde dehydrogenase B (AldB, GenBank: 948104) and aldehyde dehydrogenase H (AldH, GenBank: 8183735) gene were individually amplified by PCR from *E. coli* BL21 genomic DNA using primers FeaB-*Xba*I/FeaB-*Nhe*I–*Bam*HI, AldB-*Xba*I/AldB-*Sac*I–*Bam*HI and AldH-*Xba*I/AldH-*Nhe*I–*Bam*HI separately, and individually inserted into pET28a(+) to give pDG3, pDG4 and pDG5. The *Xba*I–*Xho*I fragment of FeaB, AldB or AldH from pDG3, pDG4 or pDG5 was individually inserted into *Nhe*I and *Xho*I sites of pDG30 to give pDG6, pDG7 and pDG8. The *Xba*I–*Xho*I fragment of ARO8 from pDG2 was inserted into *Nhe*I and *Xho*I sites of pDG8 to give pDG9. The sequences of all primers used in PCRs are listed in Table 1. Plasmids used in this study are showed in Table 2.

**Table 1:** Primers used in this study

Primer name	Sequence (5′–3′)
FeaB-*Xba*I	AACTCTAGATTTAAGAAGGAGATATAATGACAGAGCCGCATGTAGCAG
FeaB-*Nhe*I–*Bam*HI	ACAGGATCCGCTAGCTTAATACCGTACACACACCGACTTAGTTT
AldB-*Xba*I	ATCTCTAGATTTAAGAAGGAGATATAATGACCAATAATCCCCCTTCAGC
AldB-*Sac*I–*Bam*HI	TGTGAGCTCGGATCCTCAGAACAGCCCCAACGGTT
AldH-*Xba*I	ATCTCTAGATTTAAGAAGGAGATATAATGAATTTTCATCATCTGGCTTACTG
AldH-*Nhe*I–*Bam*HI	TCAGGATCCGCTAGCTCAGGCCTCCAGGCTTATCC
ARO8-*Xba*I	AACTCTAGATTTAAGAAGGAGATATAATGATGACTTTACCTGAATCAAAAGACTTTTC
ARO8-*Xho*I	CCGCTCGAGCTATTTGGAAATACCAAATTCTTCGTATAA

**Table 2:** Plasmids used in this study

Plasmids	Replication origin	Overexpressed genes	Resistance	Source
pDG2	pBR322	P_T7_: *aro8*	Kan	This study
pDG3	pBR322	P_T7_: *feaB*	Kan	This study
pDG4	pBR322	P_T7_: *aldB*	Kan	This study
pDG5	pBR322	P_T7_: *aldH*	Kan	This study
pDG6	pBR322	P_T7_: *kdc* and *feaB*	Kan	This study
pDG7	pBR322	P_T7_: *kdc* and *aldB*	Kan	This study
pDG8	pBR322	P_T7_: *kdc* and *aldH*	Kan	This study
pDG9	pBR322	P_T7_: *kdc*, *aldH* and *aro8*	Kan	This study

### Shake flask cultures to heterologous expression PAA in *E. coli*

Plasmids containing different genes were transformed into *E. coli* K-12 MG1655 strain respectively to get recombinant strains. Overnight cultures inoculated from single colonies were used to inoculate shake flasks containing M9 medium with 20 g/L glucose as previously described by Guo et al. (2015), and shaken at 30 °C as above. The cells were induced at OD600 0.6–0.8 with 0.1 mM IPTG. Samples were taken during the course of the cultures for total wax esters analyses described below.

### GC/MS analysis of PAA produced in *E. coli*

Cell cultures were harvested and prepared for wax esters using a previously published method (Guo et al. 2015). GC/MS analysis was performed with a DB-5 capillary column. The following temperature program was applied: 100 °C for 3 min, an increase of 15 °C/min to 240 °C. Quantification was done by using benzoic acid as internal standard.

## Results

### Construction of PAA biosynthetic pathway from glucose in *E. coli*

**Fig. 2:**
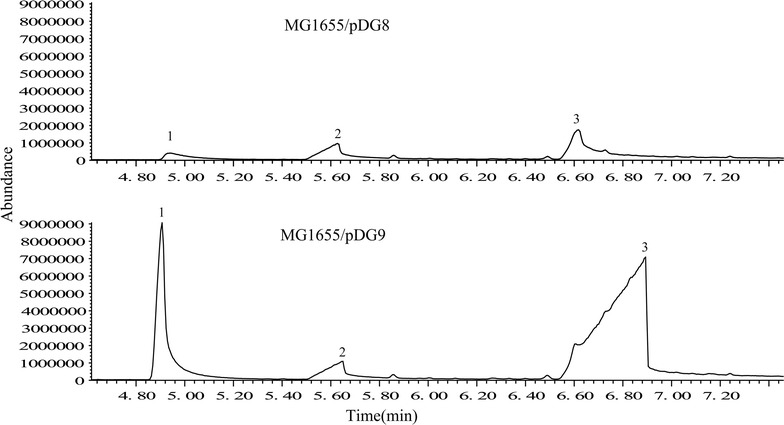
The GC/MS result of PAA from the extract of the recombinant strain MG1655/pDG8 and MG1655/pDG9 in shake flasks for 28 h. Identified substances: *1* phenethyl alcohol; *2* benzoic acid (used as internal standard, 40 mg/L); *3* phenylacetic acid (PAA)

The precursor substrate phenylpyruvate is an intermediates of shikimate pathway in *E. coli*. In this work, we overexpressed a keto acid decarboxylase (KDC) gene from *S. cerevisiae* YPH499 to enhance phenylacetaldehyde synthesis from phenylpyruvate. Then it was identified that whether several candidate genes (*feaB*, a*ldB* and *aldH*) possess aldehyde dehydrogenase activity to catalyze phenylacetaldehyde into PAA in *E. coli*. The peak of PAA was only observed on the gas chromatogram in the sample from the extract of the recombinant MG1655/pDG8 strain harboring gene *aldH* (Fig. 2), which means AldH could oxidase phenylacetaldehyde to PAA while FeaB and AldB couldn’t. Hence, our study suggests that AldH may be a efficient phenylacetaldehyde dehydrogenase for oxidation of phenylacetaldehyde to PAA. On the other hand, the peak of PAA is relatively low in GC/MS map with a titer of 49.5 ± 1.27 mg/L (Table 3), which means it is possible that phenylpyruvate from glucose is relatively low in expanded shikimate pathway (Gallardo et al. 2008). Hence we need to find another way to produce more phenylpyruvate, thus to get higher production of PAA.

**Table 3:** PAA production in engineered strain with M9 medium with or without 1.0 g/L of l-phenylalanine in shake flasks for 28 h

Production (mg/L)	The engineered *E. coli* strains		
	MG1655/pDG8	MG1655/pDG9	RARE/pDG9
PAA	49.5 ± 1.27	425.8 ± 15.41	772.9 ± 26.80

All experiments were performed in triplicate and error bars show SD

### Construction of the PAA biosynthesis pathway from l-phenylalanine in *E. coli*

There are two strategies to increase phenylpyruvate availability to improve the production of PAA. A strategy is metabolically engineered *E. coli* strain to strengthen shikimate pathway. Another strategy is transamination of l-phenylalanine to phenylpyruvate by aminotransferase. Several groups have demonstrated the biosynthesis of 2-phenylethanol from l-phenylalanine with a high titer by transamination (Kim et al. 2014; Yin et al. 2015).

The gene ARO8 encode aminotransferase was amplified from *S cerevisiae* YPH499 and introduced to the aldH and KDC PAA production system. And addition of l-phenylalanine as the substrate into this new PAA biosynthesis system with heterogenous expression of ARO8, aldH and KDC gene in an engineered strain MG1655*/*pDG9 was identified whether PAA yield has changed. From the GC/MS result, PAA production was up to 425.8 ± 15.41 mg/L PPA with the molar yield of 0.52 moL/moL (Table 3; Fig. 2), which demonstrates that the conversation rate of PAA from l-phenylalanine is higher than that from glucose.

**Fig. 3:**
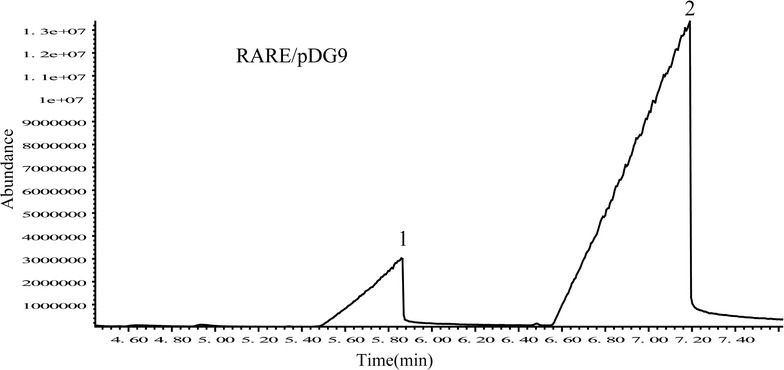
The GC/MS result of PAA from the extract of the recombinant strain RARE/pDG9 in shake flasks for 28 h. Identified substances: *1* benzoic acid (used as internal standard, 100 mg/L); *2* phenylacetic acid (PAA)

However, in the process of producing PAA in *E. coli*, it is general accompanied with the production of 2-phenylethanol converted from phenylacetaldehyde by phenylacetaldehyde reductase. Kristala concluded that RARE strain knocked out of phenylacetaldehyde reductase could prevent phenylacetaldehyde from being reduced into phenethyl alcohol (Kunjapur et al. 2014). In this study, pDG9 was transformed into *RARE* strain to get recombinant RARE/pDG9 strain. This strain produced up to 772.9 ± 26.80 mg/L PPA from 1 g/L l-phenylalanine with the molar yield of 0.94 moL/moL (Table 3; Fig. 3), which indicates that knock out of phenylacetaldehyde reductase is effective for improvement of PAA production.

## Discussion

Phenylacetic acid (PAA) is a class of important compounds represented by several industries and its demand is very large. And the approaches of PAA production have been studied by more and more researcheres in recent years (Kishore et al. 1976; Krings et al. 1996; Groot and Bont 1998). With the rapid development of the domestic spices, pharmaceuticals, pesticides and other industries, the demand of PAA will further increase. Although some methods of chemical synthesis for PAA production have lots of problems including harmful and high corrosive nature, difficulties in handling and work up procedure and their disposal today, they are the primary ways of producing PAA so far.

Recombinant microorganisms are sustainable alternative for the production of chemicals like PAA (Gavrilescu and Chisti 2005). Oelschlägel et al. recently heterologously expressed a synthetic styC membrane gene in *E. coli* BL21(DE3) pLysS for whole cell biocatalyst for the production of PAA from styrene with a conversion rate ~85% (Oelschlägel et al. 2015). PAA, as an intermediate in the catabolite pathway of phenylalanine, could also be produced from transamination of phenylalanine, decarboxylation of phenylpyruvate, and subsequent oxidation of phenylacetaldehyde. However, phenylacetaldehyde synthesis would be hindered due to the conversion of aldehydes to phenethyl alcohol. A reduced aromatic aldehyde reduction (RARE) *E. coli* K-12 MG1655 strain whose three genes that encode aldo–keto reductases and three genes that encode alcohol dehydrogenases have been deleted, got the aromatic aldehydes as end products could be accumulated in *E. coli* (Kunjapur et al. 2014).

In this study, a promising PAA biosynthetic pathway was constructed by using a RARE *E. coli* K-12 MG1655 strain as the host for heterologous expression of aminotransferase ARO8, keto acid decarboxylase KDC and oxyreductase AldH. In this construct pathway in engineered *E. coli*, the PAA is mainly derived from phenylalanine (Fig. 1) and the conversion rate is as high as 94%. The result of PAA conversion rate (94%) in our study is higher than that (85%) of Oelschlägel’s study (2015) and the process in this study is much simpler, which have many advantages in the industry application, and microbial production of PAA.

The aim of this research was to identify if it were possible to generate a distinct biological gateway for the production of PAA. We have designed a new microbial biosynthetic pathway to produce PAA from phenylalanine. Rather than using glucose as substrates, phenylalanine has superiority in improvement of PAA yield. One of the most important characteristics of this approach is that there is little dissipation of aldehydes to its corresponding 2-phenylethanol alcohol due to the RARE strain. This work demonstrates that the selection of appropriate substrate for PAA biosynthesis is a feasible method for enhancing PAA production. Future efforts to further increase PAA production via microbial metabolic engineering, such as selection of applicable and low-cost substrate, may be accomplished through overproduction of the appropriate metabolites as substrate for incorporation by some genes.

## Abbreviations

PAA: phenylacetic acid
ARO8: aminotransferase
KDC: keto acid decarboxylase
AldH: aldehyde dehydrogenase H
FeaB: phenylacetaldehyde dehydrogenase
AldB: aldehyde dehydrogenase B

## References

[CR1] Agapakis CM, Boyle PM, Silver PA (2012). Natural strategies for the spatial optimization of metabolism in synthetic biology. Nat Chem Biol.

[CR2] Alonso-Gutierrez J, Chan R, Batth TS, Adams PD, Keasling JD, Petzold CJ, Lee TS (2013). Metabolic engineering of *Escherichia coli* for limonene and perillyl alcohol production. Metab Eng.

[CR3] Chen Y, Nielsen J (2013). Advances in metabolic pathway and strain engineering paving the way for sustainable production of chemical building blocks. Curr Opin Biotechnol.

[CR4] Cook SD, Nichols DS, Smith J, Chourey PS, McAdam EL, Quittenden L, Ross JJ (2016). Auxin biosynthesis: are the indole-3-acetic acid and phenylacetic acid biosynthesis pathways mirror images?. Plant Physiol.

[CR5] Dongamanti A, Gadiparthi R, Redamala R, Anireddy J, Vantikommu J (2012). Convenient synthesis of phenolic esters of *o*-bromo-substituted phenylacetic acids. Der Pharm Chem.

[CR6] Duan J, Jiang J, Gong J, Fan Q, Jiang D (2000). Synthesis of phenylacetic acid by carbonylation. J Mol Catal A Chem.

[CR7] Gallardo E, De Schutter DP, Zamora R, Derdelinckx G, Delvaux FR, Hidalgo FJ (2008). Influence of lipids in the generation of phenylacetaldehyde in wort-related model systems. J Agric Food Chem.

[CR8] Gavrilescu M, Chisti Y (2005). Biotechnology—a sustainable alternative for chemical industry. Biotechnol Adv.

[CR9] Gilligan T, Yamada H, Nagasawa T (1993). Production of S-(+)-2-phenylpropionic acid from (R, S)-2-phenylpropionitrile by the combination of nitrile hydratase and stereoselective amidase in *Rhodococcus equi* TG328. Appl Microbiol Biotechnol.

[CR10] Giroux A, Nadeau C, Han Y (2000). Synthesis of phenylacetic acids under rhodium-catalyzed carbonylation conditions. Tetrahedron Lett.

[CR27] Groot MNN, de Bont JA (1998). Conversion of phenylalanine to benzaldehyde initiated by an aminotransferase in *Lactobacillus plantarum*. Appl Environ Microbiol.

[CR12] Guo D, Pan H, Li X (2015). Metabolic engineering of *Escherichia coli* for production of biodiesel from fatty alcohols and acetyl-CoA. Appl Microbiol Biotechnol.

[CR11] Guo D, Zhang L, Pan H, Li X (2017) Metabolic engineering of *Escherichia coli* for production of 2-phenylethylacetate from l-phenylalanine. Microbiologyopen e00486–n/a. doi:10.1002/mbo3.486 10.1002/mbo3.486 PMC5552962 28436122

[CR13] Gupta S, Marko MG, Miller VA, Schaefer FT, Anthony JR, Porter JR (2015). Novel production of terpenoids in *Escherichia coli* and activities against breast cancer cell lines. Appl Biochem Biotechnol.

[CR14] Ho KK, Weiner H (2005). Isolation and characterization of an aldehyde dehydrogenase encoded by the aldB gene of *Escherichia coli*. J Bacteriol.

[CR15] Huang H, Xia C, Xie P (2014a) Process for synthesizing phenylacetic acid by carbonylation of toluene

[CR16] Huang LF, Wang ZH, Chen SL (2014). Hypericin: chemical synthesis and biosynthesis. Chin J Nat Med.

[CR17] Huang JF, Liu ZQ, Jin LQ, Tang XL, Shen ZY, Yin HH, Zheng YG (2016). Metabolic engineering of *Escherichia coli* for microbial production of l-methionine. Biotechnol Bioeng.

[CR18] Jo JE, Raj SM, Rathnasingh C, Selvakumar E, Jung WC, Park S (2008). Cloning, expression, and characterization of an aldehyde dehydrogenase from *Escherichia coli* K-12 that utilizes 3-hydroxypropionaldehyde as a substrate. Appl Microbiol Biotechnol.

[CR19] Kim B, Cho BR, Hahn JS (2014). Metabolic engineering of *Saccharomyces cerevisiae* for the production of 2-phenylethanol via Ehrlich pathway. Biotechnol Bioeng.

[CR20] Kishore G, Sugumaran M, Vaidyanathan CS (1976). Metabolism of DL-(±)-phenylalanine by *Aspergillus niger*. J Bacteriol.

[CR21] Koma D, Yamanaka H, Moriyoshi K, Ohmoto T, Sakai K (2012). Production of aromatic compounds by metabolically engineered *Escherichia coli* with an expanded Shikimate pathway. Appl Environ Microbiol.

[CR22] Krings U, Hinz M, Berger RG (1996). Degradation of [2 H] phenylalanine by the basidiomycete *Ischnoderma benzoinum*. J Biotechnol.

[CR23] Kunjapur AM, Tarasova Y, Prather KL (2014). Synthesis and accumulation of aromatic aldehydes in an engineered strain of *Escherichia coli*. J Am Chem Soc.

[CR24] Li S, Nishimura Y, Matsuda F, Ishii J, Kondo A (2016). Overexpressing enzymes of the Ehrlich pathway and deleting genes of the competing pathway in *Saccharomyces cerevisiae* for increasing 2-phenylethanol production from glucose. J Biosci Bioeng.

[CR25] Milne JE, Storz T, Colyer JT, Thiel OR, Seran MD, Larsen RD, Murry JA (2012). Cheminform abstract: iodide-catalyzed reductions: development of a synthesis of phenylacetic acids. Cheminform.

[CR26] Nielsen J, Fussenegger M, Keasling J, Lee SY, Liao JC, Prather K, Palsson B (2014). Engineering synergy in biotechnology. Nat Chem Biol.

[CR28] Oelschlägel M, Heiland C, Schlömann M, Tischler D (2015). Production of a recombinant membrane protein in an *Escherichia coli* strain for the whole cell biosynthesis of phenylacetic acids. Biotechnol Rep.

[CR29] Rabinovitch-Deere CA, Oliver JW, Rodriguez GM, Atsumi S (2013). Synthetic biology and metabolic engineering approaches to produce biofuels. Chem Rev.

[CR30] Somers E, Ptacek D, Gysegom P, Srinivasan M, Vanderleyden J (2005). *Azospirillum brasilense* produces the auxin-like phenylacetic acid by using the key enzyme for indole-3-acetic acid biosynthesis. Appl Environ Microbiol.

[CR31] Sosedov O, Baum S, Bürger S, Matzer K, Kiziak C, Stolz A (2010). Construction and application of variants of the pseudomonas fluorescens EBC191 arylacetonitrilase for increased production of acids or amides. Appl Environ Microbiol.

[CR32] Wang J, Niyompanich S, Tai YS, Wang J, Bai W, Mahida P, Gao T, Zhang K (2016). Engineering of a highly efficient *Escherichia coli* strain for mevalonate fermentation through chromosomal integration. Appl Environ Microbiol.

[CR33] Yin S, Zhou H, Xiao X, Lang T, Liang J, Wang C (2015). Improving 2-phenylethanol production via Ehrlich pathway using genetic engineered *Saccharomyces cerevisiae* strains. Curr Microbiol.

